# The mediating role of family intimacy: negative emotions and resilience in adolescents with depressive disorders

**DOI:** 10.3389/fpsyt.2025.1606543

**Published:** 2025-10-08

**Authors:** Chunlan Li, Chaona Shang, Yuge Wang, Lina Yang, Miao Pan

**Affiliations:** ^1^ The Second Affiliated Hospital of Xinxiang Medical University, Xinxiang, Henan, China; ^2^ Henan Collaborative Innovation Center of Prevention and Treatment of Mental Disorder, The Second Affiliated Hospital of Xinxiang Medical University, Xinxiang, Henan, China; ^3^ Brain Institute, Henan Academy of Innovations in Medical Science, Zhengzhou, Henan, China; ^4^ University of Leeds, Leeds, United Kingdom

**Keywords:** depressive disorders, adolescent patients, family intimacy, negative emotions, psychological resilience

## Abstract

**Objective:**

To explore the relationship between family intimacy, negative emotions and psychological resilience in adolescent inpatients with depressive disorders.

**Methods:**

The family intimacy, adaptability scale, adolescent psychological resilience scale and positive and negative emotion scale were used to investigate 215 adolescents with depressive disorders who were hospitalized through convenience sampling method.

**Results:**

①0; There was a significant positive correlation between family intimacy and psychological resilience in adolescents with depressive disorders (*r* = 0.456, *P* < 0.01); ①1; There was a significant negative correlation between negative emotions and psychological resilience in adolescents with depressive disorders (r = -0.543, *P* < 0.01); ①2; There was a significant negative correlation between negative emotions and family intimacy in adolescents with depressive disorders (*r* = -0.260, *P* < 0.01); ①3; Negative emotions in adolescents with depressive disorders not only had a significant negative predictive effect on psychological resilience (*β* = -0.878, *t* = -9.445, *P* < 0.001), but also had a significant negative predictive effect on psychological resilience through family intimacy (*β* = -0.736, *t* = -8.275, *P* < 0.001) Negative emotions in adolescents with depressive disorders had a significant negative predictive effect on family intimacy (*β* = -0.338, *t* = -3.936, *P* < 0.001); ①4; Family intimacy played a partial mediating role between negative emotions and psychological resilience, and the mediating effect accounted for 16.17% of the total effect.

**Conclusion:**

Reducing negative emotions and strengthening family intimacy can improve the level of psychological resilience of patients, thereby reducing self-injury and suicide in adolescents with depressive disorders.

## 1 Introduction

The World Health Organization (WHO) predicts that by 2030, depression will become the world’s leading burden of disease (1). In recent years, the prevalence of depression among adolescents worldwide has significantly increased and shows group differences. According to national data in the Philippines, the prevalence of moderate to severe depression symptoms increased from 9.6% in 2013 to 20.9% in 2021, with particularly significant increases among women, sexual minorities, and low-income adolescents (2). A study in the United States shows that 59.5% of depressed adolescents belong to the anxiety depression subtype, which is characterized by a higher risk of suicide and functional impairment (3). With the rapid development of society, adolescents are not only facing the pressure of physiological changes, but also the pressure of learning and interpersonal relationships, which can easily lead to the generation of negative emotions in adolescents, and then self-injury and suicide behavior, which brings a heavy burden to individuals, families and society (4).

Negative emotions reflect an individual’s stress and unpleasant emotions, including depression, anxiety, tension, etc., which can significantly negatively affect psychological resilience (5). Psychological resilience is a process in which individuals can quickly adjust and adapt in the face of great pressure, trauma and threats. Psychological resilience is beneficial to the recovery and prognosis of patients with mental illness (6). Family intimacy is also closely related to adolescent negative emotions and psychological resilience. Family intimacy is the emotional connection between individuals and families, an indicator of close relationships between family members and a good family atmosphere (7). Family intimacy can also significantly affect psychological resilience. Psychological resilience can help them maintain a good balance with family members, improve self-injury and suicide behavior in adolescents with depression (8). In previous studies, the relationship between each variable and other variables was mostly explored, but the relationship between the three variables was lacking. Therefore, this study proposes the following hypotheses: ①0; Negative emotions are significantly negatively correlated with psychological resilience; ①1; Family intimacy is significantly positively correlated with psychological resilience; ①2; Family intimacy plays a partial mediating role between negative emotions and psychological resilience. To sum up, this study plans to study the relationship between family intimacy, negative emotions and psychological resilience in adolescent hospitalized patients with depressive disorders, and focuses on analyzing the mediating role of family intimacy between negative emotions and psychological resilience.

## 2 Objects and methods

### 2.1 Objects

Adolescent patients with depressive disorders who were hospitalized in the Second Affiliated Hospital of Xinxiang Medical University in Henan Province from July to December 2023 were selected by convenience sampling method. Enrollment criteria: ①0; Meet the diagnostic criteria for depressive disorder in the 10th edition of the International Classification of Diseases (ICD-10); ①1; Age 11–18 years. ①2; Sign the informed consent form and participate voluntarily. Exclusion criteria: ①0; Concomitant with other mental illnesses; ①1; Severe excitement, impulsiveness and incompatibility. The average age of the survey respondents was (14.64 ± 1.73) years old; 40 boys (18.60%) and 175 girls (81.40%); 20 primary school students (9.30%), 118 junior high school students (54.88%), 60 high school students (27.91%), and 17 high school students (7.91%) and above. According to multiple linear regression analysis, the minimum sample size should be 5 to 10 times that of the independent variable. Considering the special circumstances such as invalid questionnaires or missing samples in the survey, the sample size should be increased by 10% (9). A total of 235 questionnaires were distributed, and 215 invalid questionnaires were recovered after excluding incomplete responses, with an effective collection rate of 91.48%. All patients and main caregivers in this study were aware of the content of the study and signed informed consent forms.

### 2.2 Method

#### 2.2.1 The positive and negative affect schedule

PANAS was performed as previously describe (9), with good reliability and validity, this study selects the negative emotion sub-scale of this scale, including 10 entries, using Liker 5-level scoring method (1 = almost none, 5 = extremely many), the higher the score, the more negative emotions. Cronbach’s α coefficient of negative emotions in this study is 0.884.

#### 2.2.2 Resilience scale for adolescents

READ was used to assess the severity of adolescents with depressive disorders (10). The scale contains 39 items, and aims to assess the protective resources of personal competence, social competence, structured style, family cohesion, and social resources to understand stress adaptation. Using the Liker 5-point scoring method (0 = completely inconsistent, 5 = completely consistent), the higher the score, the higher the individual’s psychological resilience level. In this study, the scale’s Cronbach’s alpha coefficient was 0.833.

#### 2.2.3 Family intimacy scale

A sub-scale of the Family Intimacy and Adaptability Scale is adopted, namely the Family Intimacy Scale (11), which has good reliability and validity. The scale contains a total of 16 items and adopts Liker 5-level score (1 = no, 5 = always). The higher the score, the higher the individual family intimacy level. In this study, the scale’s Cronbach’s α coefficient was 0.782.

### 2.3 Statistical processing

Statistical analysis of the data was carried out by SPSS 25.0 statistical software, including common method deviation test, descriptive statistical analysis, t-test, Pearson correlation analysis, and process 3.3 plug-in model 4 was used for mediation effect test.

## 3 Results

### 3.1 Common method deviation test

In order to avoid the influence of common method bias, the Harman single factor test was used to test the common method bias. The results showed that there were 13 factors with characteristic root greater than 1, and the variation explained by the first factor was 17.69% (40% below the critical value). Therefore, there was no serious common method bias in this study.

### 3.2 Descriptive statistical analysis and correlation analysis of each variable

T-test was used to analyze the differences in gender and only child, and the results showed that there were no significant differences in gender and only child in negative emotions, psychological resilience and family intimacy (*P* = 0.239-0.501, *P* = 0.267-0.531).

The correlation analysis results are shown in **Table 1**. From the table, it can be seen that negative emotions are significantly negatively correlated with psychological resilience. The more negative emotions, the lower the level of psychological resilience; negative emotions are significantly negatively correlated with family intimacy; family intimacy and psychological resilience are significantly positively correlated.

**Table 1:** Correlation analysis between negative emotions and family intimacy and psychological resilience (*r*).

Variable	$\bar{\text{x}}$ ± s	Negative emotions	Family intimacy	Psychological resilience
negative emotions	29.50 ± 9.02	1		
family intimacy	61.51 ± 11.71	-0.260**	1	
psychological resilience	74.28 ± 14.57	-0.543**	0.456**	1

**:*P*<0.01.

### 3.3 Test of the mediating effect of family intimacy between negative emotions and psychological resilience

In order to explore the mechanism of family intimacy between negative emotions and psychological resilience, SPSS 25.0 and Process 3.3 were used to test the mediating effect of family intimacy, using family intimacy as a mediating variable, negative emotions as a predictor, and psychological resilience as a dependent variable for mediating effect analysis.

**Figure 1:**
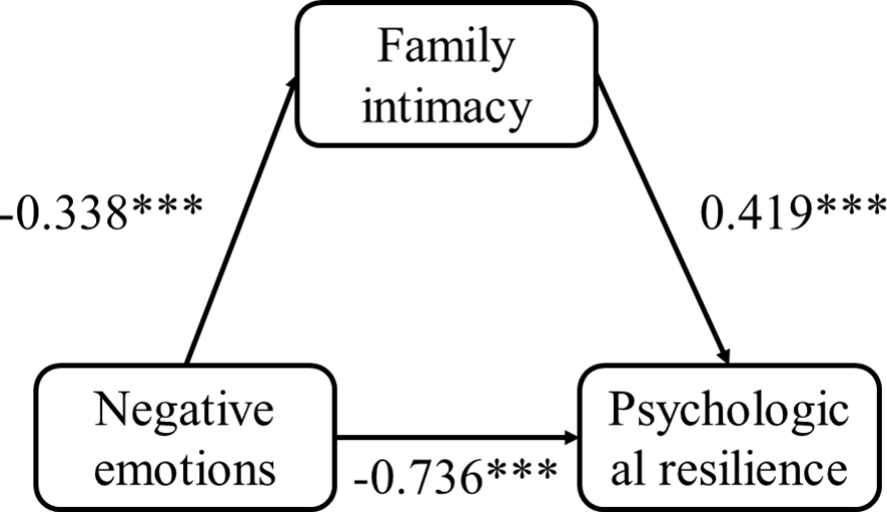
Family intimacy between negative emotions and psychological resilience in adolescent inpatients with depressive disorders mediation model. ***: P < 0.001.

Before conducting the mediation effect test, it is necessary to estimate the parameters of the regression equation in the model. As shown in **Table 2**, negative emotions have a significant negative predictive effect on psychological resilience, negative emotions have a significant negative effect on family intimacy. Negative emotions can significantly predict psychological resilience, and family intimacy can significantly predict psychological resilience. The mediation model is depicted in **Figure 1**.

**Table 2:** Regression analysis among negative emotions, family intimacy, and psychological resilience.

Dependent variable	Predictor variable	R	R^2^	*F*	*β*	*t*
psychological resilience	negative emotions	0.543	0.295	89.202***	-0.878	-9.445***
family intimacy	negative emotions	0.260	0.068	15.489***	-0.338	-3.936***
psychological resilience	negative emotions	0.633	0.401	70.954***	-0.736	-8.275***
	family intimacy				0.419	6.119***

***:*P*<0.001.

Bootstrap was used to test the mediating role of family intimacy in the relationship between negative emotions and psychological resilience, and 5,000 retractions were extracted from the original data source. As shown in **Table 3**, the total effect value is -0.878, and the 95% confidence interval is [-1.060, -0.694], excluding 0, which proves that the total effect is significant. The direct effect is -0.736, and the 95% confidence interval is [-0.911, -0.561], excluding 0, which proves that the direct effect is significant, and the direct effect accounts for 83.83% of the total effect. The indirect effect is -0.142, and the 95% confidence interval is [-0.237, -0.065], excluding 0, which proves that the indirect effect is significant, that is, the mediating effect is significant. Family intimacy plays a mediating role between negative emotions and psychological resilience, and the mediating effect accounts for 16.17% of the total effect.

**Table 3:** Results of the mediating effect test of family intimacy.

Effect type	Effect value	Boot standard error	Boot 95% CI		Relative effect (%)
			Lower bound	Upper limit	
total effect	-0.878	0.092	-1.060	-0.694	
direct effect	-0.736	0.089	-0.911	-0.561	83.83
indirect effect	-0.142	0.044	-0.237	-0.065	16.17

## 4 Discussions

### 4.1 Analysis of the status quo of adolescent inpatients with depressive disorders, negative emotions, family intimacy, and psychological resilience

The results of this study showed that the negative mood score of adolescent inpatients with depressive disorders was (29.50 ± 9.02) points, which was similar to the results that reported previously (12). It shows that the negative emotions of adolescent inpatients with depressive disorders are more obvious. The possible reason is that the physical and psychological development of adolescents with depression is not mature enough in the process of growth, which easily leads to psychological imbalance, poor emotional regulation ability, and is easily affected by negative emotions. Research suggests that patients often have strong negative emotions before self-injury suicide, and emotional imbalance is the main reason for self-injury suicide (13). Therefore, it is recommended to pay attention to the emotional changes of adolescents, strengthen communication, understand their psychological needs and feelings, and guide positive emotions. In this survey, the family intimacy score of adolescent hospitalized patients with depressive disorders was (61.51 ± 11.71) points, which was lower than that of middle school students without depressive disorders. It is consistent with the results of previous studies (14). The survey found that adolescents who grew up in a good family environment have closer parent-child relationships, and bad family relationships are more likely to make adolescents lose control of their emotions and take extreme ways to solve problems when facing difficulties (15). Therefore, communication between family members should be strengthened to cultivate a harmonious parent-child relationship, which will help reduce adolescent self-injury and suicide/impulsive violence. In this study, the psychological resilience score of adolescents with depressive disorders was (74.28 ± 14.57), which was lower than that reported previously (16). This suggests that adolescents with depressive disorders lack effective coping styles and adaptability to adversity and difficulties. The survey found that individuals with high psychological resilience are more likely to adopt active coping strategies to solve problems in difficult situations, reduce the adverse effects of stress, and thus reduce self-harm and suicidal behavior (17). Therefore, attention should be paid to the level of psychological resilience of adolescents, guide them to correctly face difficulties and setbacks in growth, and take positive coping methods.

### 4.2 Correlation analysis of negative emotions, family intimacy and psychological resilience in adolescents with depressive disorders

The results of correlation analysis showed that there was a significant negative correlation between negative emotions and psychological resilience in adolescents with depressive disorders. This is also consistent with prior research (18). Negative emotions affect their psychological resilience level. The more obvious the negative emotions, the lower the psychological resilience level. The survey found that negative emotions are catalysts for self-injury or violence (19). The more negative emotions an individual experiences, the weaker the positive coping style and psychological adjustment ability. Individuals with low psychological resilience are prone to anxiety and unease about their environment, easily immersed in sadness and pain, and difficult to recover from adversity. Previous studies have shown that positive emotions and psychological resilience can cause positive behavioral effects, making them actively seek support and adopt more appropriate coping styles, thereby alleviating the negative effects of adverse events (20). Therefore, attention should be paid to the emotional state of adolescents, and timely care and guidance should be given to their negative emotions, so as to improve the level of psychological resilience and help adolescents adopt good coping strategies when coping with difficulties. In addition, the results of this study show that there is a significant positive correlation between family intimacy and psychological resilience, that is, the better the family intimacy, the higher the level of psychological resilience of adolescents. Research suggests that family environment is closely related to adolescents’ mental health (21). A good family environment helps adolescents to build mature personalities and adopt more active coping strategies in the face of negative and adverse events. Individuals with good family intimacy are more likely to develop positive and optimistic psychological characteristics in the process of growth. Mental toughness can correct family functions to a certain extent, encourage them to play a positive role, achieve the purpose of regulating negative emotions, and avoid self-injury and violence. Finally, this study found that the negative emotions of adolescents with depressive disorders were negatively correlated with family intimacy, that is, the more negative emotional experiences, the lower the family intimacy. This is consistent with the results of previous studies (22). Adolescents with self-harm and suicide have more negative emotional experiences, incomplete understanding of things, inability to correctly identify and deal with setbacks, deviation from parents’ cognition, prone to family conflicts, and difficulty in maintaining good parent-child relationships. Therefore, it is recommended that we pay attention to the positive effects of family intimacy, strengthen parent-child communication, enhance trust between family members, and create a warm and understanding family environment, so as to improve the negative emotions of adolescents.

### 4.3 The mediating role of family intimacy between negative emotions and psychological resilience in adolescents with depressive disorders

The results of this study show that the family intimacy of adolescents with depressive disorders has a significant positive effect on psychological resilience, which is consistent with the findings that reported previously (11, 23). Family intimacy reflects the emotional connection between individuals and families and reflects the close relationship between family members. Individuals with high family intimacy have fewer family conflicts and are more likely to receive social support during their growth. They are able to actively seek help, respond flexibly and adapt to adversity (24). The survey found that the close emotional connection between family members contributes to the development of individual psychological resilience (25). And psychological resilience also helps to modify family function, help individuals obtain good growth resources from the family, and help regulate the impact of family environment on adolescents’ self-injury and suicide behaviors (26). At the same time, the results of the study showed that family intimacy plays a partial mediating role between negative emotions and psychological resilience in adolescents with depressive disorders, accounting for 16.17% of the total effect. Improving the family intimacy of adolescents is beneficial to help them feel more warmth in the family, enabling timely and effective communication of information and emotions, providing adolescents with more care and support, and helping to relieve negative emotions such as depression and anxiety (25, 27). In addition, it is also beneficial to improve the stress response ability of adolescents, enabling them to give up extreme self-harm and suicide methods and adopt a more proactive way to cope with difficulties and stress, thereby improving their psychological resilience level. It is recommended to emphasize the positive role of family function, call for strengthening parent-child communication and emotional exchange, closely monitoring adolescent emotional changes, guiding positive emotions, cultivating good psychological quality, enhancing adolescent stress coping ability, and improving mental toughness.

In addition, this study found that the proportion of female adolescent patients with depression was significantly higher than that of males (81.40% *vs*. 18.60%). This gender disparity may be attributed to factors such as women’s greater tendency toward rumination, hormonal fluctuations, and sociocultural pressures (28–30). Previous research indicates that women are more sensitive to family dynamics, where a dysfunctional family environment is more likely to trigger emotional issues, while male depression often manifests as externalizing behaviors (31). These findings suggest that clinical interventions should account for gender differences—for instance, enhancing emotion-regulation training for females while focusing on family support and behavioral management for males to improve overall intervention outcomes.

## Data Availability

The raw data supporting the conclusions of this article will be made available by the authors, without undue reservation.
